# 2-Hydroxyethyl Methacrylate/Gelatin/Alginate Scaffolds Reinforced with Nano TiO_2_ as a Promising Curcumin Release Platform

**DOI:** 10.3390/polym15071643

**Published:** 2023-03-25

**Authors:** Marija M. Babić Radić, Vuk V. Filipović, Jovana S. Vuković, Marija Vukomanović, Tatjana Ilic-Tomic, Jasmina Nikodinovic-Runic, Simonida Lj. Tomić

**Affiliations:** 1 University of Belgrade, Faculty of Technology and Metallurgy, Karnegijeva 4, 11000 Belgrade, Serbia; 2 University of Belgrade, Institute of Molecular Genetics and Genetic Engineering, Vojvode Stepe 444a, 11000 Belgrade, Serbia; 3 Advanced Materials Department, Jožef Stefan Institute, Jamova Cesta 39, 1000 Ljubljana, Slovenia

**Keywords:** nanoTiO_2_, 2-hydroxyethyl methacrylate, gelatin, alginate, hybrid scaffolds, curcumin release platform

## Abstract

The idea of this study was to create a new scaffolding system based on 2-hydroxyethyl methacrylate, gelatin, and alginate that contains titanium(IV) oxide nanoparticles as a platform for the controlled release of the bioactive agent curcumin. The innovative strategy to develop hybrid scaffolds was the modified porogenation method. The effect of the scaffold composition on the chemical, morphology, porosity, mechanical, hydrophilicity, swelling, degradation, biocompatibility, loading, and release features of hybrid scaffolds was evaluated. A porous structure with interconnected pores in the range of 52.33–65.76%, favorable swelling capacity, fully hydrophilic surfaces, degradability to 45% for 6 months, curcumin loading efficiency above 96%, and favorable controlled release profiles were obtained. By applying four kinetic models of release, valuable parameters were obtained for the curcumin/PHEMA/gelatin/alginate/TiO_2_ release platform. Cytotoxicity test results depend on the composition of the scaffolds and showed satisfactory cell growth with visible cell accumulation on the hybrid surfaces. The constructed hybrid scaffolds have suitable high-performance properties, suggesting potential for further in vivo and clinical studies.

## 1. Introduction

The topic of developing advanced biomaterials that play an irreplaceable and highly effective role in improving the healing process and tissue regeneration has been a very intense scientific field for decades. In this field of research, hydrogels are considered leading materials that can provide many benefits and excellent outcomes for healing [1,2,3,4,5]. Hydrogels have proven as remarkable biomaterials for designing versatile scaffolds with multipurpose functions in biomedical applications due to their potential to mimic the functional performances of native tissue. Their exceptional ability is to provide a highly hydrated environment suitable for the settlement, support, and growth of new cells due to the presence of bioactive agents that improve the regeneration process through controlled dosing of the bioactive agent [6,7,8].

The creation of efficient polymeric systems for the controlled release of bioactive agents is inextricably linked with the design of potent scaffolds embedded with biomolecules. Therefore, such systems should exhibit advanced biological and therapeutic activities. For these purposes, both natural and synthetic origin polymers are the most used due to their superior properties for the ability to form a 3D hydrophilic structure with a favorable water/biological fluid content and to enable the population cells into their porous structures, as well as load and controlled release various bioactive agents [9]. Polymeric systems based on stimuli-sensitive hydrogels can be managed by acting to specific stimuli from the internal/external changes in the environment of cells and tissues, leading to the manipulation with cells in a controlled direction as well as controlled/targeted release of a bioactive agent(s) [10,11,12,13,14]. Hydrogels based on natural origin polymers are primarily advantageous due to their favorable biocompatibility, biodegradability, lower immunogenicity, excellent cytocompatibility as well as the potential for various functionalizations to obtain precisely defined and desired structures with adjustable physiochemical and biological properties [15,16]. Natural origin polymers, alginate and gelatin, are hydrophilic types of polysaccharides and polypeptides used to prepare hydrogel scaffolds as well as controlled delivery systems loaded with bioactive agents. For decades, alginate has been successfully used to make hydrogels due to mild conditions for gelation with a high level of productivity and profitability, resulting in highly biocompatible biomaterials [17]. Biodegradable polypeptide gelatin is obtained through the hydrolysis of collagen and is localized predominantly in the connective tissues in the body [18]. Gelatin manifests higher water-binding capacity, fine solubility in water and significantly lower immunogenicity due to the defragmenting of the triple helix and the propeptides degradation [19]. Hydrogels based on gelatin possess specific temperature-sensitive behavior, good biocompatibility, biodegradability, and antimicrobial activity, making them suitable for biomedical applications [20,21,22,23]. Some of the scientific strategies use polymers of natural origin to create suitable scaffolds with improved biological activity by loading and releasing a bioactive agent that refers to their combination with synthetic monomers and/or inorganic components. An extraordinary example of a synthetic monomer widely used for biomedical applications is 2-hydroxyethyl methacrylate (HEMA). Hydrogels based on HEMA (PHEMA) have been studied for a long time in biomedical fields due to their superior qualities, including biocompatibility, soft and flexible structure, superior hydrophilicity, optical transparency, hydrophilic nature, and good mechanical strength. However, since the biodegradability of scaffolds is an important property for biomedical engineering applications using HEMA, without modification/functionalization its use is considerably limited [24,25,26,27].

Inorganic compounds have been used to obtain special classes of biomaterials for hydrogel scaffolds capable of loading and releasing bioactive agents. The incorporation of a rigid inorganic compound may enhance the mechanical strength and, in some cases, the biodegradability of biomaterials. In recent years, as an important inorganic compound, titanium(IV) oxide (TiO_2_) has become the focus of scaffold design, with improved biological activity due to non-toxicity, high biocompatibility, bioactivity, ability to support adhesion and proliferation of cells, excellent mechanical stability, and availability in large quantities [28,29].

The goal of this study was to project new hybrid scaffolds for efficient bioactive agent loading and release to improve the healing and tissue regeneration process. Hybrid scaffolds based on 2-hydroxyethyl methacrylate, gelatin, alginate, and TiO_2_ nanoparticles were obtained by the simple and efficient modified porogenation method. Hybrid scaffolds were tested by morphology, porosity, mechanical strength, hydrophilicity, swelling capacity, in vitro degradation, and in vitro cytotoxicity assay in interaction with normal human fibroblast cells. Curcumin was selected as the model drug for in vitro release study from hybrid scaffolds. The in vitro controlled curcumin release process was monitored. Based on the four release models that were used to fit the experimental results for the curcumin/PHEMA/G/A/TiO_2_ hybrid scaffold platform, the parameters and models that best describe the release phenomena were determined. 

## 2. Materials and Methods

### 2.1. Materials

Synthetic monomer 2-hydroxyethyl methacrylate (HEMA, purity 99%) and natural origin polymers-alginate (A, powder, biomedical polymer) and gelatin (G, type A powder, bioreagent, suitable for cell culture) were purchased from Sigma–Aldrich, St. Louis, MO, USA. N,N′-methylene bisacrylamide (BIS, purity 99%, Sigma–Aldrich, St. Louis, MO, USA) was used as a crosslinking agent of HEMA. Pluronic F-172 (reagent grade 98%), ammonium persulfate (APS, reagent grade 98%) and N,N,N′,N′-tetramethylene diamine (TEMED, 98%) were obtained from Sigma–Aldrich, St. Louis, MO, USA and used in all polymerizations as a foaming stabilizer, initiator, and activator, respectively. Porogen agent-sodium bicarbonate, inorganic compound titanium(IV) oxide nanoparticles (rutile nanopowder < 100 nm particle size, 99.5% trace metals basis) (TiO_2_) and active agent-curcumin (from Curcuma longa (Tumeric), powder, quality level 200, assay > 65%, Sigma–Aldrich, St. Louis, MO, USA). N-Ethyl-N′-(3-dimethyl aminopropyl)carbodiimide hydrochloride (EDC, 98.0%) as the crosslinking agent of gelatin was purchased from Sigma–Aldrich, St. Louis, MO, USA. RPMI-1640 medium and supplements for cell proliferation as well as 3-(4,5-dimethylthiazol-2-yl)-2,5-diphenyltetrazolium bromide (MTT) reduction assay components were purchased from Sigma–Aldrich, St. Louis, MO, USA. Potassium hydrogen phosphates (KH_2_PO_4_ and K_2_HPO_4_, Sigma–Aldrich) were used for buffer preparations. All experiments were performed using lab-produced, ultra-distilled water.

### 2.2. Hybrid Scaffolds Synthesis

Hybrid scaffolds based on synthetic monomer HEMA, polymers-gelatin (G), and alginate (A) and TiO_2_ nanoparticles were synthesized using a modified porogenation method varying gelatin/alginate ratio (Table 1). HEMA, 2.5% solution of BIS and 10% solution of Pluronic were added in a glass tube and stirred at 50 °C for 1 h, then gelatin and alginate previously dissolved in deionized water were added and stirred for 45 min. Then, the solutions of 10% APS and 10% TEMED were added to the reaction mixture and heated at 63 °C. When the reaction mixture reaches 63 °C, the mixture of TiO_2_ nanoparticles and porogen was added quickly and vigorously vortexed to distribute porogen as well as TiO_2_ uniformly throughout the polymeric hydrogel network. Samples containing gelatin were immersed in an EDC solution for 2 h for crosslinking reaction. Hydrogels were cut into discs (8 mm) and washed with deionized water for 7 days to remove unreacted components. Samples were placed in a deep freezer (−80 °C) and lyophilized (−55 °C) for further characterization.

### 2.3. Hybrid Scaffolds Characterization

#### 2.3.1. Fourier Transform Infrared Spectroscopy

The chemical structure of hybrid scaffolds was determined by FTIR spectra recorded over the wavelength range of 700–4000 cm^−1^ on an FTIR Nicolet 6700 (Thermo FisherScientific, Waltham, MA, United States) diamond crystal spectrometer with attenuated total reflectance (ATR) sampling technique.

#### 2.3.2. Scanning Electron Microscopy (SEM)

The morphology of hybrid scaffolds was observed by a scanning electron microscope EOL JSM-7600F. The lyophilized samples were previously sputtered with gold by BAL-TEC SCD 005 and dried in a VC 50 SalvisLab Vacucenter vacuum chamber.

#### 2.3.3. Porosity Measurements

The porosity of hybrid scaffolds was calculated by the solvent replacement method, using the true and bulk density of the hybrid scaffolds as well as the density obtained by the Archimedes method, where glycerol (ρ_0_ = 1.2038 g/cm^3^) was used as a wetting medium [30].

#### 2.3.4. Mechanical Properties Testing

The mechanical properties of the hybrid scaffolds were determined using compression tests. The tests were carried out using a Galdabini Quasar 50 testing machine (Cardano Al Campo, Varese, Italy) on cylindrically shaped specimens that were accurately measured for their dimensions (diameter and height) before testing. The specimens were compressed uniaxially with a 100-N load cell at a crosshead speed of 0.5 mm/min at room temperature. The elastic moduli were determined from the slope of the linear elastic region of the stress-strain curves.

#### 2.3.5. In Vitro Swelling Study

The swelling capacity of hybrid scaffolds was monitored in a simulated physiological conditions-phosphate buffer of pH 7.40 at 37 °C, calculating the degree of swelling by gravimetric method [31,32,33]. All experiments were performed in triplicate.

#### 2.3.6. Water Contact Angle Measurements

The static water contact angle measurement was analyzed on a Theta Lite-Biolin Scientific Contact angle meter in a measuring range from 0 to 180 deg. and accuracy ±0.1 deg., ±0.01 mN/m, applying the sessile drop method by putting approximately 1 μL drop of MilliQ water on the scaffold’s surface. All experiments were performed in triplicate.

#### 2.3.7. In Vitro Degradation Study

In vitro degradation studies were conducted by the gravimetric method. Samples were placed into a 10 mL buffer pH of 7.40 at 37 °C for 3 and 6 months, removed from the buffer and dried at room temperature to constant weight. Degradation was expressed as a percentage of sample weight loss [34]. All experiments were performed in triplicate.

### 2.4. In Vitro Cytotoxicity Assay

Cytotoxic/antiproliferative activity of the hybrid scaffolds was determined using human lung fibroblasts (MRC5 cells) and the standard MMT assay [30]. MRC5 cells were incubated in RPMI-1640 medium containing FBS, penicillin, and streptomycin at 37 °C for 24 h. They were then treated with 50%, 25%, and 12.5% (*v*/*v*) scaffold extracts and 200 µg/mL of the ground and filtered scaffold and incubated for an additional 48 h. Control cultures were treated with a growth medium. Cell proliferation was calculated by measuring absorbance at 540 nm using the Tecan Infinite 200 Pro Multiple Reader (Tecan Group, Mannedorf, Switzerland) [35,36]. Images were acquired using the CKX41 phase contrast microscope (Olympus, Tokyo, Japan). All experiments were performed in triplicate.

### 2.5. In Vitro Controlled Curcumin Release Study

#### 2.5.1. Drug Loading and Entrapment Efficiency of Hybrid Scaffolds

Curcumin was selected as a bioactive agent and loaded into hybrid scaffolds using the swelling-diffusion method. The hybrid scaffolds were immersed in 5 mL of curcumin solution to reach an equilibrium swelling state [36]. The potential drug loading (DL) and.0 entrapment efficiency (EE) of the hybrid scaffolds were calculated as previously described [33].

#### 2.5.2. In vitro Curcumin Release Study

Hybrid scaffolds loaded with curcumin were placed in a basket stirrer containing 15 mL of release medium (phosphate buffer pH of 8.00) at 37 ± 0.5 °C. UV/VIS spectrophotometer (Shimadzu UV/Vis Spectrophotometer UV-1800) was used to measure the concentration of curcumin released from the hybrid scaffolds by taking the absorbance of the released media at λ_max_ value of 430 nm in determined time intervals.

#### 2.5.3. Analysis of Curcumin Transport Mechanism

To reveal the transport mechanisms and curcumin release kinetics from hybrid scaffolds, the first 60% of the in vitro obtained curcumin release data were fitted using different release kinetic models Higuchi, Ritger–Peppas, Peppas–Sahlin and Peppas–Sahlin model (where m = 0.5) [37,38,39,40].

### 2.6. Statistical Analyses

The Origin Pro 9.0 software was used to analyze data. The data are expressed as mean ± SD.

## 3. Results and Discussion

**Scheme 1:**
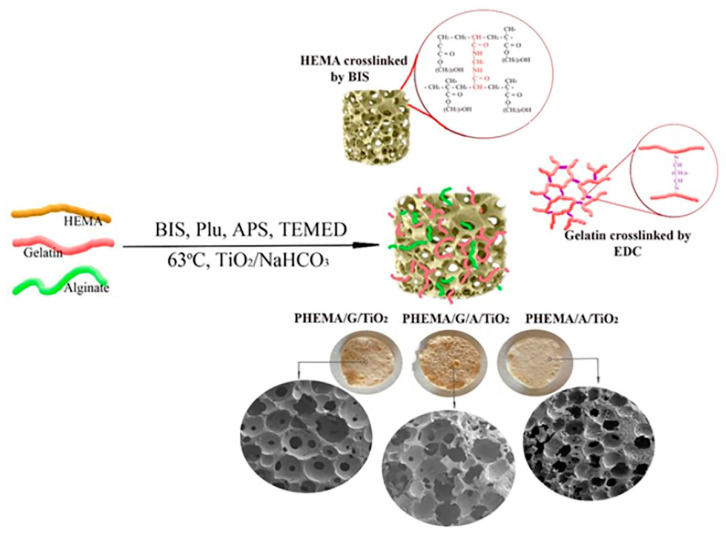
Synthesis path for the hybrid scaffolds based on PHEMA, gelatin, alginate, and TiO_2_ obtained by modified porogenation method (details: photographs of samples and their microstructures).

New hybrid scaffolds loaded with bioactive agent curcumin were prepared by modified porogenation method (monomer HEMA crosslinked by BIS (PHEMA, poly(2-hydroxyethyl methacrylate)). Gelatin was crosslinked by EDC and intertwined with linear alginate chains (Scheme 1) to create a promising curcumin release platform.

### 3.1. Structural Characteristics of Hybrid Scaffolds

**Figure 1:**
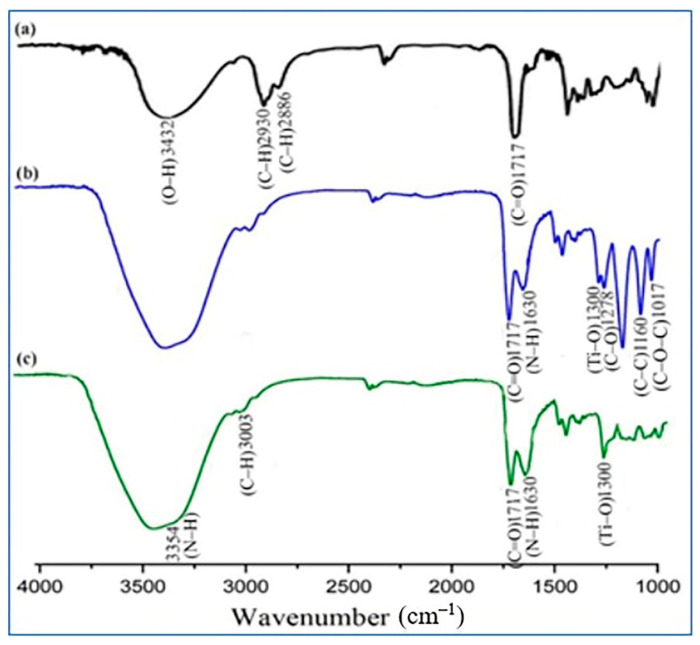
FTIR spectra of (**a**) PHEMA, (**b**) PHEMA/A/TiO_2_, and (**c**) PHEMA/G/TiO_2_ hybrid scaffolds.

The chemical structure of hybrid scaffolds based on PHEMA, gelatin, alginate, and TiO_2_ was detected by Fourier transform infrared spectroscopy (FTIR). Figure 1 shows the FTIR spectra of PHEMA and scaffolds based on PHEMA/alginate and PHEMA/gelatin reinforced with TiO_2_. The FTIR spectra of all samples confirmed the presence of PHEMA component by typical peaks for O–H stretching vibration at around 3432 cm^−1^, C–H stretching vibrations at 2930 and 2886 cm^−1^, and strong C=O vibration at 1717 cm^−1^. The FTIR spectrum of PHEMA/A/TiO_2_ showed peaks for PHEMA, peaks of alginate C–O stretching at 1278 cm^−1^, C–C stretching at 1160 cm^−1^ and C–O–C stretching at 1017 cm^−1^ [41,42]. Typical peaks for gelatin found in the FTIR spectrum of PHEMA/G/TiO_2_ at 3354 cm^−1^, 3003 cm^−1^, and 1630 cm^−1^ related to N–H stretching vibration, C–H stretching vibration, and N–H stretching vibration for the amide II, indicating the presence of gelatin in the scaffold [43]. The greater intensity of the peaks in the spectra of PHEMA/A/TiO_2_ and PHEMA/G/TiO_2_ around 3500 cm^−1^ corresponded to the stretching vibration of the hydroxyl group O–H of the TiO_2_ and the appearance of the peaks at 1630 cm^−1^ and 1300 cm^−1^ corresponded to bending modes of water Ti–OH and Ti–O modes, confirming an embedding of TiO_2_ in the samples [42,43].

### 3.2. Morphology of Hybrid Scaffolds

The porous morphology is a leading property for the scaffold design and capability for loading bioactive agent and releasing it. This porous morphology is imperative for scaffolding biomaterial to provide host-cell population, neovascularization, cell migration, and flow of nutrients and oxygen for cell proliferation and tissue regrowth [44,45,46,47]. Among that, the size of the pores and their interconnectivity is also essential for cell-penetration depth within the interior of the scaffold. To facilitate cell migration and proliferation, the size of pores in scaffolding biomaterial must be larger than the physical size of cells. Therefore, the precise control of the porous morphology of the scaffolding biomaterial can promote suitable biomaterial and cell interactions [44,45,46,47]. Porous scaffolds have a great capacity for drug loading and controlled release. Interconnected pores of different sizes are suitable for the loading of various types of bioactive agents, including molecules with larger dimensions such as proteins and growth factors.

**Figure 2:**
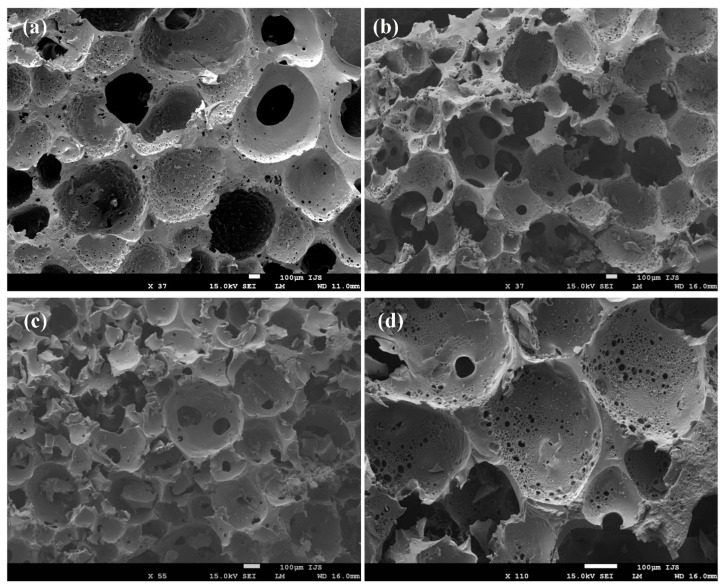
SEM micrographs of a cross-section of (**a**) PHEMA/G/TiO_2_, (**b**) PHEMA/70G/30A/TiO_2_, (**c**) PHEMA/50G/50A/TiO_2_ and (**d**) PHEMA/A/TiO_2_ hybrids (the bar is 100 μM).

Since the goal of this study is to design new scaffolding biomaterials with great potential to load and release bioactive agents, the morphology of the hybrid scaffolds was investigated by SEM. The obtained SEM micrographs of the cross-section for the hybrid scaffolds (Figure 2) show well-defined, highly porous morphology with interconnected pores mainly in spherical shapes due to the preparation procedure by spherical particles of porogen, which is also recognized as a beneficial way for good interconnectivity in the porous scaffolds as well as for the tuning of mechanical properties of scaffolds [48]. The main pattern of the morphology of the hybrid scaffolds is “numerous small pores within a macropore” that forms an environment enriched with pores of desirable sizes (micro- and macropores) convenient for a bioactive agent with different dimensions loading as well as various types of cells. Additionally, obtained micrographs show that increased content of alginate in the hybrid scaffolds tended to form an increased number of smaller pores compared to scaffolds based on PHEMA, gelatin and TiO_2_, indicating that the morphology of the hybrids can be modulated by varying their content. Thus, the morphology of the hybrid scaffolds can be easily adapted to the specific requirements of their final biomedical applications.

### 3.3. Porosity of Hybrid Scaffolds

**Figure 3:**
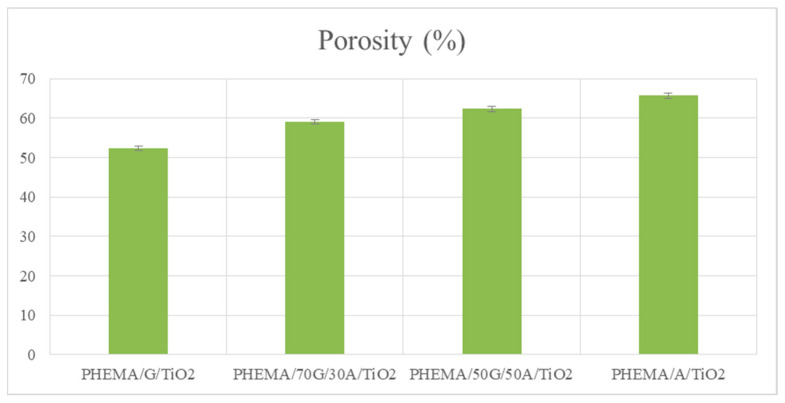
Porosity values for PHEMA/G/A/TiO_2_ hybrid scaffolds.

The porosity offered by these types of hybrid scaffolds results in improved tissue binding ability due to the high surface-to-volume ratio inducing bioactivity. Interconnected pores provide a scaffold for tissue growth in the scaffold matrix and anchor them to the surrounding tissue, preventing micro-movements which in turn increase further tissue growth. Interconnected porosity is also a source of oxygen and nutrient supply and helps in vascularization and waste removal. Porosity is an essential property for assessing the internal structure and free spaces of scaffolds and is expressed as the percentage of free space within the total volume of biomaterial construct. It was noticed that inadequate porosity of the scaffold leads to inappropriate cell function inside the scaffold (penetration, distribution, and regrowth). The appropriate porosity of scaffolds for successful cell populations is above 50% and depends on the type of cells [45,46,47,48,49]. The porosity values of obtained PHEMA/G/A/TiO_2_ hybrid scaffolds are in the range of 52.33% to 65.76% (Figure 3). The influence of the composition of hybrid scaffolds on their porosity is evident. Hybrid scaffolds containing a higher content of gelatin have a lower porosity due to crosslinked gelatin. The introduction of linear alginate chains (PHEMA/70G/30A/TiO_2_ and PHEMA/A/TiO_2_ samples) into the hybrids leads to an increase in porosity because segments of the linear chains affect the structures of the polymeric networks of PHEMA/gelatin in terms of increasing the free space within the scaffold. Hybrid scaffolds containing alginate have a porosity above 60%, which is very suitable for their biomedical applications.

### 3.4. Swelling Properties of Hybrid Scaffolds

**Figure 4:**
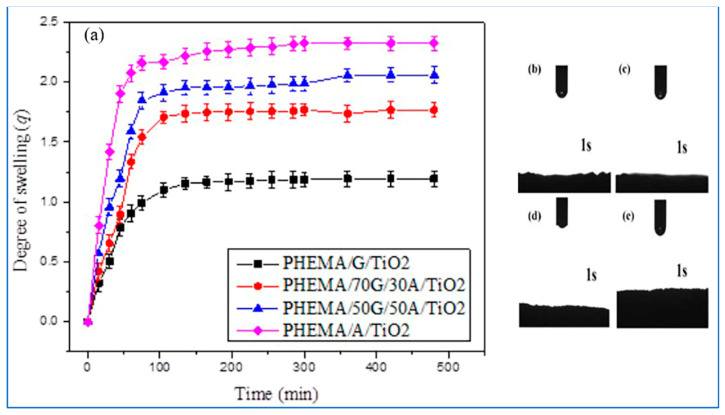
(**a**) Swelling profiles for PHEMA/G/TiO_2_, PHEMA/70G/30A/TiO_2_, PHEMA/50G/50A/TiO_2_, PHEMA/A/TiO_2_; Water contact angle measurements in 1 s of (**b**) PHEMA/G/TiO_2_, (**c**) PHEMA/70G/30A/TiO_2_, (**d**) PHEMA/50G/50A/TiO_2_, (**e**) PHEMA/A/TiO_2_ hybrid scaffolds.

The swelling capacity of hydrogels is an essential characteristic for evaluating the potential of hydrogel scaffolds for the design of a platform for the efficient release of bioactive agents for biomedical and tissue engineering applications and considering its influence on the transport of nutrients and oxygen within the scaffold [49]. Swelling capacity is affected by numerous factors, such as crosslinking level, functional groups of the polymeric chains, nature of polymers, porosity, pore size as well as interconnectivity of pores. The swelling capacity of the hybrid scaffolds was monitored in vitro in a buffer of pH 7.40 at 37 °C to simulate the physiological microenvironment. Obtained swelling results are presented in Figure 4a as the value of the degree of swelling (q) vs. time and are in the range of 1.20 to 2.25. As shown in Figure 4a, the initial fast swelling of the hybrid scaffolds was achieved because of the highly hydrophilic nature of the hybrids and interconnected porous microstructure. It is also evident that swelling capacity depends on the chemical composition of the hybrid scaffolds. Hybrid scaffolds with higher content of gelatin show the lowest degree of swelling (q of 1.20) due to crosslinked gelatin. The values of q increase by decreasing the content of gelatin in hybrid scaffolds due to lower crosslinked gelatin content. 

### 3.5. Hydrophilicity of Hybrid Scaffolds

The hydrophilic/hydrophobic nature of the surface of biomaterial is a significant property that affects the interaction and response of cells and can be analyzed by the water surface contact angle. In the case where obtained water surface contact angle is above 90°, the surface of the scaffold is considered hydrophobic while when the obtained angle is less than 90° degrees, the surface is considered hydrophilic. Versatile scientific studies confirmed that cells thrive greater on scaffolding biomaterials with hydrophilic surfaces which promote better cell adhesion and proliferation [50,51,52]. It was observed that the adhesion of osteoblasts decreases when the surface contact angle increased from 0° to 106°, while the fibroblasts have maximum adhesion when the contact angle is less than 80° [52]. Obtained results of water surface contact angle measurements performed at 0 and 1 s for the PHEMA/G/A/TiO_2_ hybrid scaffolds are presented in Figure 4b–e. The results revealed fully hydrophilic surfaces of all samples. Water fully wetted the surface of the PHEMA/G/A/TiO_2_ hybrid scaffolds and the water drop immediately disappeared after placing it on the hybrid scaffold surface. This behavior classifies the PHEMA/G/A/TiO_2_ hybrid scaffolds as highly convenient scaffolding biomaterials for efficient adhesion, proliferation, and differentiation of various types of cells as well as for tissue engineering applications.

### 3.6. In Vitro Degradation Behavior of Hybrid Scaffolds

**Figure 5:**
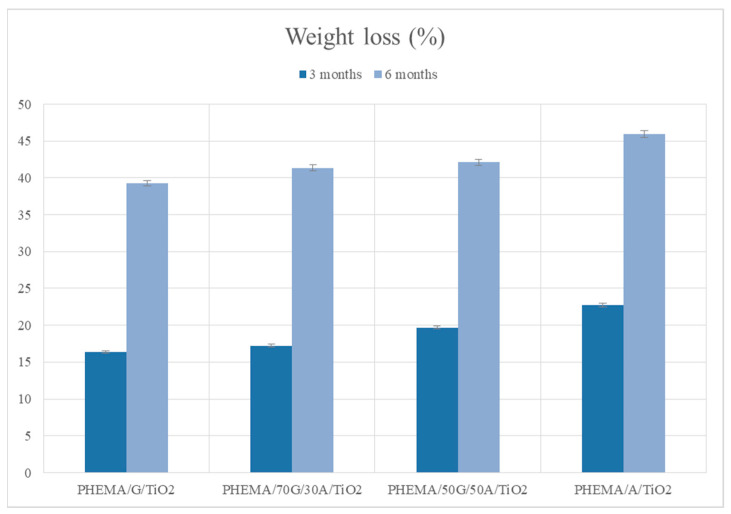
Degradation of PHEMA/G/TiO_2_, PHEMA/70G/30A/TiO_2_, PHEMA/50G/50A/TiO_2_, PHEMA/A/TiO_2_ hybrid scaffolds for three and six months.

The degradability of scaffolding biomaterials is also a significant property for biomedical applications to enable cell and/or tissue regeneration in vitro/in vivo conditions [53,54]. During degradation, a free space is created inside the scaffold, which is used for the settlement of cells and the further successful process of tissue regeneration. This property is controlled by biomaterial mass dissolution which takes place through processes like hydrolysis, photolysis, separation, or their combination [55]. Degradation of biomaterials guided by the hydrolytic process is imperative for tissue engineering applications since the primary mechanism of the degradation of scaffold inside the body is hydrolysis. Moreover, physiological conditions can be simulated by adjusting the temperature to 37 °C and the pH value. During the degradation process, various properties of the scaffold as well as its efficiency can be evaluated [56]. In vitro degradation behavior of the PHEMA/G/A/TiO_2_ hybrid scaffolds was monitored in simulated physiological conditions (37 °C and pH of 7.40) for 3 and 6 months. The obtained results of degradation (Figure 5) are in the range of 16.33–22.75% after three months and 39.28–45.89% after six months, indicating dependence on the composition of hybrid scaffolds. The introduction of alginate into the hybrid‘s composition (PHEMA/G/A/TiO_2_ samples) leads to increased degradation compared to PHEMA/G/TiO_2_ scaffold. This phenomenon could be explained as an effect of linear alginate chains (which are not crosslinked) leaching out of the polymeric network. Also, the influence of hydrophilic groups from the components that build the scaffolds and the interaction with the water medium contributes to the degradation process. Therefore, the degradation behavior of the hybrids can be tuned by varying scaffold composition. Scaffold degradation data are correlated with porosity values. These results are in agreement with the finding that adjusting the gelatin crosslinking can influence the degradation of biomaterials [37,57].

### 3.7. Mechanical Properties of Hybrid Scaffolds

**Figure 6:**
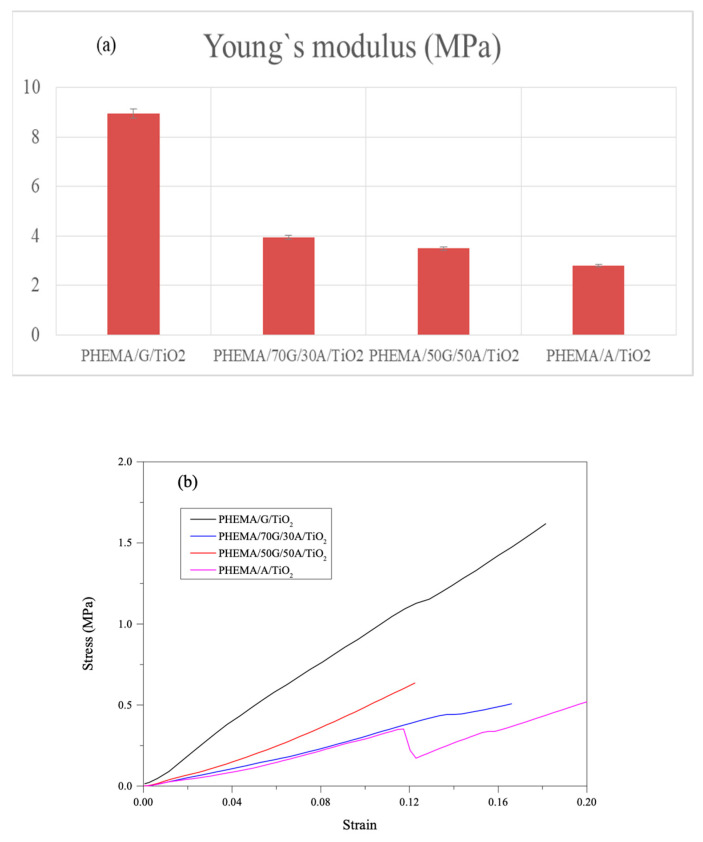
(**a**) Modulus values of PHEMA/G/TiO_2_, PHEMA/70G/30A/TiO_2_, PHEMA/50G/50A/TiO_2_, PHEMA/A/TiO_2_ hybrids; (**b**) Stress-strain curves of PHEMA/G/TiO_2_, PHEMA/70G/30A/TiO_2_, PHEMA/50G/50A/TiO_2_, PHEMA/A/TiO_2_ hybrids.

Mechanical properties of biomaterials are important requirements that provide structural functionality and durability of a scaffold. Preferably, a scaffold should be similar to the anatomy of its targeted tissue application site and should imitate the original, native structure of the targeted tissues [52,58]. Moreover, since each type of tissue realizes different functions and possesses different physiology and morphologies, the optimal requirements for mechanical properties of scaffolding biomaterials are individual to the targeted type of tissue. The mechanical properties of prepared PHEMA/G/A/TiO_2_ hybrid scaffolds were tested and obtained results of Young’s modulus are presented in Figure 6. As can be seen, obtained values of Young’s modulus are in the range of 2.81 to 8.94 MPa and show dependence on composition. The higher value of elastic modulus was obtained for the PHEMA/G/TiO_2_ hybrid scaffolds due to the higher crosslinked gelatin content and lower porosity, which contributes to the mechanical strength. The addition of linear polymer alginate to the composition of the hybrid scaffolds results in lower values of Young’s modulus, which may be suitable for soft tissue regeneration. The obtained data for the mechanical properties follow the porosity results (Figure 3), as a higher percentage of porosity (empty space inside the scaffold) leads to weaker mechanical properties [56]. Therefore, it is a wise issue to develop scaffold biomaterials with highly interconnected porous structures with optimal pore size, porosity, and mechanical strength to ensure adequate nutrients and oxygen transport and diffusion, as well as to promote cell proliferation and tissue regrowth. A specific and powerful advantage of hybrid scaffolds is the ability to precisely design parameters and functionality by adjusting the selection of components (their chemical composition) depending on the requirements of the biomedical application field.

### 3.8. In Vitro Cytotoxicity of Hybrid Scaffolds

**Figure 7:**
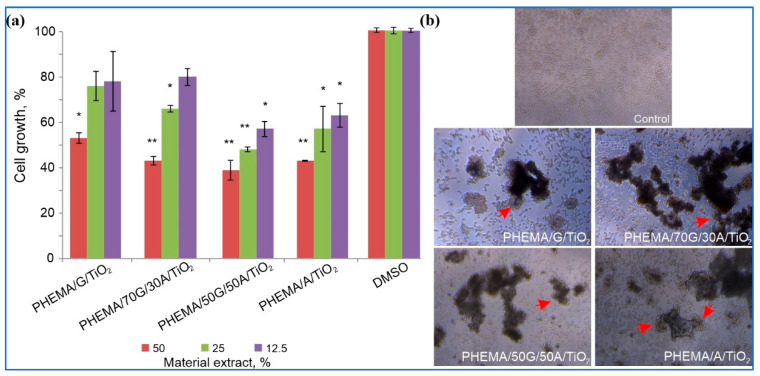
(**a**) Cell growth after 48 h of treatment with extracts of PHEMA/G/TiO_2_, PHEMA/70G/30A/TiO_2_, PHEMA/50G/50A/TiO_2_, and PHEMA/A/TiO_2_ hybrid scaffolds (results are averages of *n* = 6, error bars represent standard deviation and percentage of alive cells was compared to the DMSO-treated control using a *t*-test; * *p* ≤ 0.05, ** *p* ≤ 0.01); (**b**) Cell growth in the presence of ground materials after 48 h (red arrowheads indicate accumulation of cells on the material surface). Images were taken under a microscope at 20× magnification.

The evaluation of in vitro cytocompatible behavior of scaffolding biomaterials is a significant step for confirmation that obtained scaffolds can be used for medical purposes and not induce toxic or side effects in living organisms. The in vitro cytocompatibility of PHEMA/G/A/TiO_2_ hybrid scaffolds was evaluated in an assay on MRC5 using the MTT test. Obtained results are shown in Figure 7. PHEMA/G/TiO_2_, PHEMA/70G/30A/TiO_2_, and PHEMA/A/TiO_2_ showed favorable effects on cell growth. PHEMA/50G/50A/TiO_2_ sample has a lower impact on cell growth. The results of hybrid scaffolds’ direct contact with the cells show the cell accumulation on the material surface (Figure 7b) which confirms that prepared PHEMA/G/A/TiO_2_ hybrid scaffolds can be safely used for biomedical applications [59,60,61].

### 3.9. Drug Loading and Entrapment Efficiency for Curcumin

**Figure 8:**
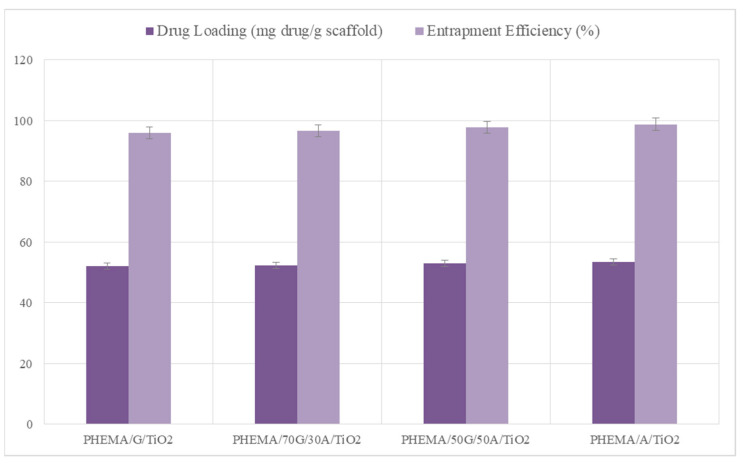
Drug loading and entrapment efficiency of PHEMA/G/TiO_2_, PHEMA/70G/30A/TiO_2_, PHEMA/50G/50A/TiO_2_, PHEMA/A/TiO_2_ hybrids.

A new generation of scaffolding biomaterials for biomedical engineering applications tends toward sophisticated scientific advances in the tissue regeneration process, not only by supporting cell proliferation but also by targeted/local delivery/release of bioactive agents essential as a natural additional support for tissue formation of targeted tissues. In that regard, the ability of PHEMA/G/A/TiO_2_ hybrid scaffolds to load and release bioactive agents was monitored in vitro in a phosphate buffer of pH 8.00 at 37 °C to simulate the physiological microenvironment. Curcumin was selected as a bioactive agent model due to its versatile biological activity (anti-inflammatory, antioxidant, antimicrobial, antiviral, and anticancer properties) but limited clinical uses due to poor water solubility and low bioavailability. Because the formulation of active agents poorly soluble in water represents a current challenge in modern pharmacy and biomedicine, this study tends to create an efficient platform for the successful formulation of a bioactive agent without the use of toxic organic solvents. Obtained data for curcumin loading (DL) and entrapment efficiency (EE) of the PHEMA/G/A/TiO_2_ hybrid scaffolds are presented in Figure 8. The dependence of DL and EE on the composition of the hybrid scaffolds and their swelling capacity is observed. Scaffolds based on PHEMA/gelatin/TiO_2_ can load the lowest amount of curcumin 52.01 mg curcumin/g scaffold because of its lower swelling capacity compared to the scaffolds based on PHEMA/A/TiO_2_ better loading capacity (a higher amount of curcumin 53.54 mg curcumin/g scaffold) due to favorable swelling performances. By introducing gelatin into the hybrid scaffolds (gelatin as component crosslinked PHEMA/G network), the swelling capacity becomes lower resulting in a reduced capacity of the polymeric network to absorb curcumin. The better swelling capacity of scaffolds facilitates the diffusion of the bioactive agent through the polymeric network and improves embedding into pores which results in higher values of DL and EE. Obtained values of EE for the hybrid scaffolds are in the range of 96.02% to 98.84%, suggesting the efficacy of the hybrids to load curcumin where desired formulation can be achieved by varying the composition of scaffolds.

### 3.10. In Vitro Curcumin Release Study

**Figure 9:**
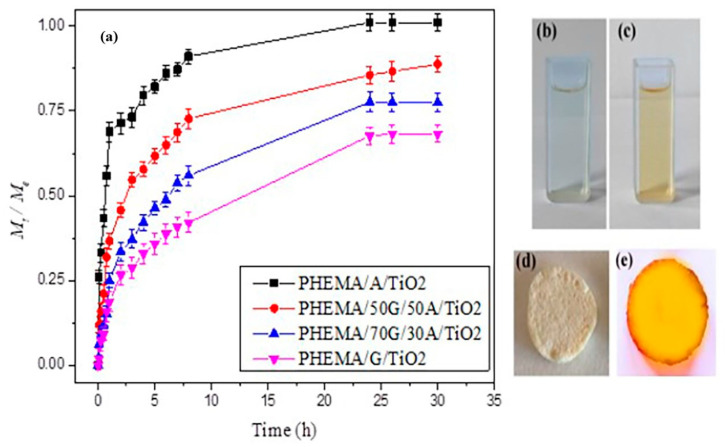
(**a**) Curcumin release profiles from PHEMA/G/A/TiO_2_ hybrid scaffolds; release medium after (**b**) 1 h and (**c**) 24 h of curcumin release; photographs of (**d**) unloaded and (**e**) curcumin loaded PHEMA/G/TiO_2_ scaffold.

The efficiency of PHEMA/G/A/TiO_2_ hybrid scaffolds as release platform wasevaluated by in vitro curcumin release study in a simulated physiological microenvironment. Obtained release profile data are presented in Figure 9a and indicate dependence on the composition of PHEMA/G/A/TiO_2_ hybrid scaffolds. As shown in Figure 9a, notable differences in the released amount of curcumin were detected for PHEMA/G/TiO_2_ and PHEMA/A/TiO_2_ samples due to significant differences in their swelling capacity. The curcumin release rate increases with increasing the alginate content which is consistent with obtained swelling data. Curcumin releases up to 24 h with a short initial “burst” period (around 2 h) for all hybrid scaffolds. It is also observed that the hybrid scaffolds loaded with a higher amount of curcumin showed faster release due to the presence of different curcumin loading levels inside scaffold pores, which facilitate the diffusivity of the bioactive agent [62].

#### Analysis of the Curcumin Transport Mechanism

To determine the mechanism of curcumin release from the hybrid scaffolds, four kinetics models were applied on obtained release data (Table 2). Calculated parameters are presented in Table 2.

To determine the mechanism and kinetics of curcumin release, various release models were applied [63,64,65,66,67,68]. To reveal the best model for the curcumin release process from the PHEMA/G/A/TiO_2_ hybrid scaffolds, the sum of the squared residuals (SSRs) and Akaike Information Criterion (AIC) was calculated and analyzed using four release kinetic models (Table 2). The model with the smallest AIC value best describes the curcumin release phenomenon from the hybrid scaffolds [69,70,71,72,73]. Obtained AIC values indicated that Peppas–Sahlin and Peppas–Sahlin when m = 0.5 models best describe the curcumin release mechanism from obtained hybrid scaffolds. An important parameter which defines the process that controls curcumin release from the hybrid scaffolds is the values of the exponent n from the Ritger–Peppas model. The calculated *n* values (*n* < 0.5) point out that the curcumin transport mechanism is Fickian diffusion for prepared PHEMA/G/A/TiO_2_ hybrid scaffolds. Additionally, to determine the contribution and synergistic effect of two different mechanisms (Fickian diffusion and polymeric chain relaxation) of curcumin release from hybrid scaffolds Peppas–Sahlin model was used. The calculated values of the kinetic constants K_1_ (define Fickian transport contribution) and K_2_ (define polymeric chains relaxation contribution) indicate that the Fickian transport is the dominant mechanism for the curcumin release from all obtained PHEMA/G/A/TiO_2_ hybrid scaffolds (K_1_ > K_2_).

## 4. Conclusions

New hybrid scaffolds based on PHEMA/G/A/TiO_2_ were synthesized by a modified porogenation method. The physicochemical testings of the PHEMA/G/A/TiO_2_ hybrid scaffolds revealed that the composition variation permits tailoring of all properties of scaffolds for different areas of biomedical applications. The porous structure with interconnected pores and tunable porosity values up to 66%, and in vitro degradation grows up to 45.89% after 6 months. Fully hydrophilic surfaces and in vitro cytotoxicity assay on MRC5 cells with visible cell accumulation on the surface of the scaffolds were obtained. Excellent curcumin loading and release performances with entrapment efficiency above 96% are powerful properties for their potential biomedical application as scaffolds with improved bioactivity. The efficient curcumin/PHEMA/G/A/TiO_2_ release platform was designed. Valuable release parameters were obtained for the curcumin/PHEMA/G/A/TiO_2_ release platform using four release kinetic models. Favorable properties of new PHEMA/G/A/TiO_2_ hybrid scaffolds for biomedical applications recommend further detailed testings with a variation of TiO_2_ fraction, as well as in vivo and clinical studies in the future research.

## Figures and Tables

**Table 1:** Composition and labels of samples.

Sample	HEMA (µL)	10% Gelatin (µL)	10% Alginate (µL)	TiO_2_ (mg)
PHEMA/G/TiO_2_	2250	2250	0	2.50
PHEMA/70G/30A/TiO_2_	2250	1575	675	2.50
PHEMA/50G/50A/TiO_2_	2250	1125	1125	2.50
PHEMA/A/TiO_2_	2250	0	2250	2.50

**Table 2:** Curcumin release data for PHEMA/G/TiO_2_, PHEMA/70G/30A/TiO_2_, PHEMA/50G/50A/TiO_2_, and PHEMA/A/TiO_2_ hybrid scaffolds fitted using: (a) Higuchi; (b) Ritger–Peppas; (c) Peppas–Sahlin; (d) Peppas–Sahlin model when m = 0.5.

Release Model	Parameters	PHEMA/A/TiO_2_	PHEMA/50G/50A/TiO_2_	PHEMA/70G/30A/TiO_2_	PHEMA/G/TiO_2_
(a) Higuchi model	*k_H_*	0.086	0.039	0.026	0.019
	*SSR*	0.007	0.001	0.014	0.964
	*AIC*	−19.21	−34.19	−55.26	−49.88
	*R* ^2^	0.976	0.965	0.985	0.974
(b) Ritger–Peppas model	*K*_1_	0.103	0.063	0.030	0.030
	*n*	0.449	0.406	0.477	0.431
	*SSR*	0.006	0.006	0.006	0.005
	*AIC*	−25.41	−36.45	−58.41	−49.94
	*R* ^2^	0.974	0.984	0.985	0.988
(c) Peppas–Sahlin model	*K*_1_	0.095	0.039	0.019	0.022
	*K*_2_	0.0170	−0.0006	−0.0001	−0.0002
	*m*	0.338	0.577	0.620	0.514
	*SSR*	0.005	0.003	0.004	0.004
	*AIC*	−26.21	−48.14	−62.14	−56.11
	*R* ^2^	0.971	0.990	0.989	0.990
(d) Peppas–Sahlin model when m = 0.5	*K*_1_	0.090	0.051	0.029	0.023
	*K*_2_	−0.0007	0.0008	0.0001	0.0001
	*SSR*	0.00333	0.00188	0.00203	0.00183
	*AIC*	−25.54	−49.46	−59.24	−52.12
	*R* ^2^	0.971	0.989	0.986	0.990

## Data Availability

Not applicable.
